# eHealth literacy, health self-efficacy, and health-promoting lifestyle among vocational college students: a latent profile and mediation analysis study

**DOI:** 10.3389/fpubh.2026.1864980

**Published:** 2026-07-15

**Authors:** Yuanyuan Ni, Wenjie Li, Xuan Ji, Yongchao Huo, Fang An, Dongming Sun

**Affiliations:** 1 Department of Infirmary, Wuxi University of Technology, Wuxi, Jiangsu, China; 2 School of Medicine, Chongqing College of Humanities, Science & Technology, Chongqing, China; 3 School of Nursing, Changzhi Medical College, Changzhi, Shanxi, China

**Keywords:** eHealth literacy, health-promoting lifestyle, latent profile analysis, self-efficacy, vocational college students

## Abstract

**Background:**

eHealth literacy (eHL) has emerged as an important competency for promoting health equity. Vocational college students are often considered a digitally vulnerable population. Understanding the heterogeneity of eHL in this population and the underlying psychological mechanisms is essential for designing effective health promotion interventions.

**Objective:**

This study aimed to identify latent profiles of eHL among vocational college students, compare differences in health-promoting lifestyle levels across profiles, and examine the mediating role of health self-efficacy.

**Methods:**

A cross-sectional survey was conducted among vocational college students from February to March 2026. The eHealth Literacy Scale, the Health Self-Efficacy Scale, and the Health-Promoting Lifestyle Profile-II were used to collect data. Latent profile analysis (LPA) was used to identify sub-groups, and bias-corrected bootstrap analyses were performed to test the mediation effects.

**Results:**

A total of 594 vocational college students were included. The mean total eHL score was 25.21 ± 5.26. Three distinct profiles were identified: Low Application-High Confidence (Profile 1, 15%), High Application-Low Critical Thinking (Profile 2, 52%), and High eHealth Literacy (Profile 3, 33%). For health-promoting lifestyle, students in Profiles 2 and 3 scored significantly higher than those in Profile 1 (both *p* < 0.001), while no significant difference was found between Profile 2 and Profile 3 (*p* > 0.05). Mediation analysis showed that health self-efficacy partially statistically mediated the association between eHL profile and health-promoting lifestyle when comparing Profile 1 with Profile 2 (effect = 0.463, standard error [SE] = 0.091, 95% confidence interval [CI] 0.345, 0.586) and Profile 1 with Profile 3 (effect = 0.697, SE = 0.126, 95% CI 0.464, 0.963). The proportion mediated increased from 43.62% for the transition from low to moderate eHL to 64.37% for the transition from low to high eHL.

**Conclusion:**

eHL among vocational college students was heterogeneous, and health self-efficacy was statistically tested as a significant mediator in the association between eHL profiles and health-promoting lifestyle, though causal inference is limited by the cross-sectional design. These findings suggest that vocational college students should not be treated as a homogeneous population in health promotion practice. Instead, person-centered interventions should be tailored to specific eHL profiles.

## 1 Introduction

The rapid evolution of digital technologies has significantly transformed healthcare delivery (1, 2). These developments may improve access to health services, reduce health inequalities, and promote progress toward universal health coverage (3). However, a well-recognized “digital paradox” remains: populations that could benefit most from digital health advances often face the greatest barriers to access and utilization (4, 5). A key important reason for this paradox is variation in individuals’ eHealth literacy (eHL) (5, 6). eHL is defined as the ability to engage with digital technologies in effective, safe, and helpful ways to achieve health goals (7). It has emerged as an important competency for promoting health equity and narrowing the digital divide (8, 9).

The university years represent a pivotal period for shaping students’ health-promoting lifestyles (HPL), which have lasting effects on long-term health outcomes, including the alleviation of subhealth conditions (10) and reduced all-cause mortality (11). Notably, nearly 42% of global cancer deaths in 2023 were attributable to modifiable lifestyle factors (12), highlighting the value of promoting healthy behaviors from early adulthood as a cost-effective public health strategy. Despite this importance, vocational college students, who account for nearly half of the higher education population in China, remain underrepresented in the literature (13). In general, they exhibit lower levels of HPL than students in comprehensive universities (14). Previous studies have shown a moderate correlation between eHL and health-related behaviors (15, 16). Individuals with higher levels of eHL had a 3.4-fold lower risk of unfavorable health-promoting lifestyle behaviors than those with lower levels (14). Currently, person-centered approaches to improving eHL have been increasingly recommended (5, 17). However, previous studies often focus on the average level of eHL and treat college students as a homogeneous group, thereby overlooking the heterogeneity within this population (18).

To address this issue, our study adopted a person-centered approach using latent profile analysis (LPA) to identify distinct eHL profiles among vocational college students. In addition, it is necessary to examine the psychological mechanism linking eHL profiles to HPL. According to Social Cognitive Theory, individual behavior is influenced not only by skills but also by cognitive mediators (19). Within this framework, health self-efficacy (HSE) (20), defined as the belief in one’s capability to perform health-related actions, may act as a critical mediator between eHL and HPL (21, 22). However, it remains unclear whether this mediating role operates similarly across different eHL profiles.

Therefore, this study aimed to (1) identify latent profiles of eHL among vocational college students; (2) examine differences in HPL and HSE across these profiles; and (3) test the mediating role of HSE in the association between eHL profiles and HPL. These findings may provide an empirical basis for designing targeted digital health interventions for vocational college students and contribute to efforts to promote health equity in the digital era.

## 2 Methods

### 2.1 Study design

A cross-sectional survey was conducted at a university of technology in Wuxi, China, from February to March 2026. A multistage stratified cluster random sampling method was used. Students were first stratified by college (eight colleges) and academic year (3 years). Within each college-year stratum, the number of classes to be sampled was determined proportionally based on student enrollment to ensure representativeness across strata. A total of 24 classes (approximately 40 students per class) were randomly selected using a computer-generated random number method, with classes serving as the sampling clusters. All eligible students in the selected classes were invited to participate. Data were collected during scheduled class meetings. With the assistance of head teachers and class monitors, students completed the questionnaires in a supervised classroom setting. Participation was voluntary, and students were informed that they could decline participation without penalty.

### 2.2 Ethical considerations

The study protocol was reviewed and approved by the Ethics Committee of Changzhi Medical College (Approval No.: RT2026001). Electronic informed consent was obtained from all participants before survey completion. For participants younger than 18 years, informed consent was additionally obtained from their parents or legal guardians. On the first page of the online survey, participants were informed of the study purpose, the voluntary nature of participation, the anonymous and confidential handling of their data, and that the data would be used for research purposes only.

### 2.3 Participants

**Figure 1:**
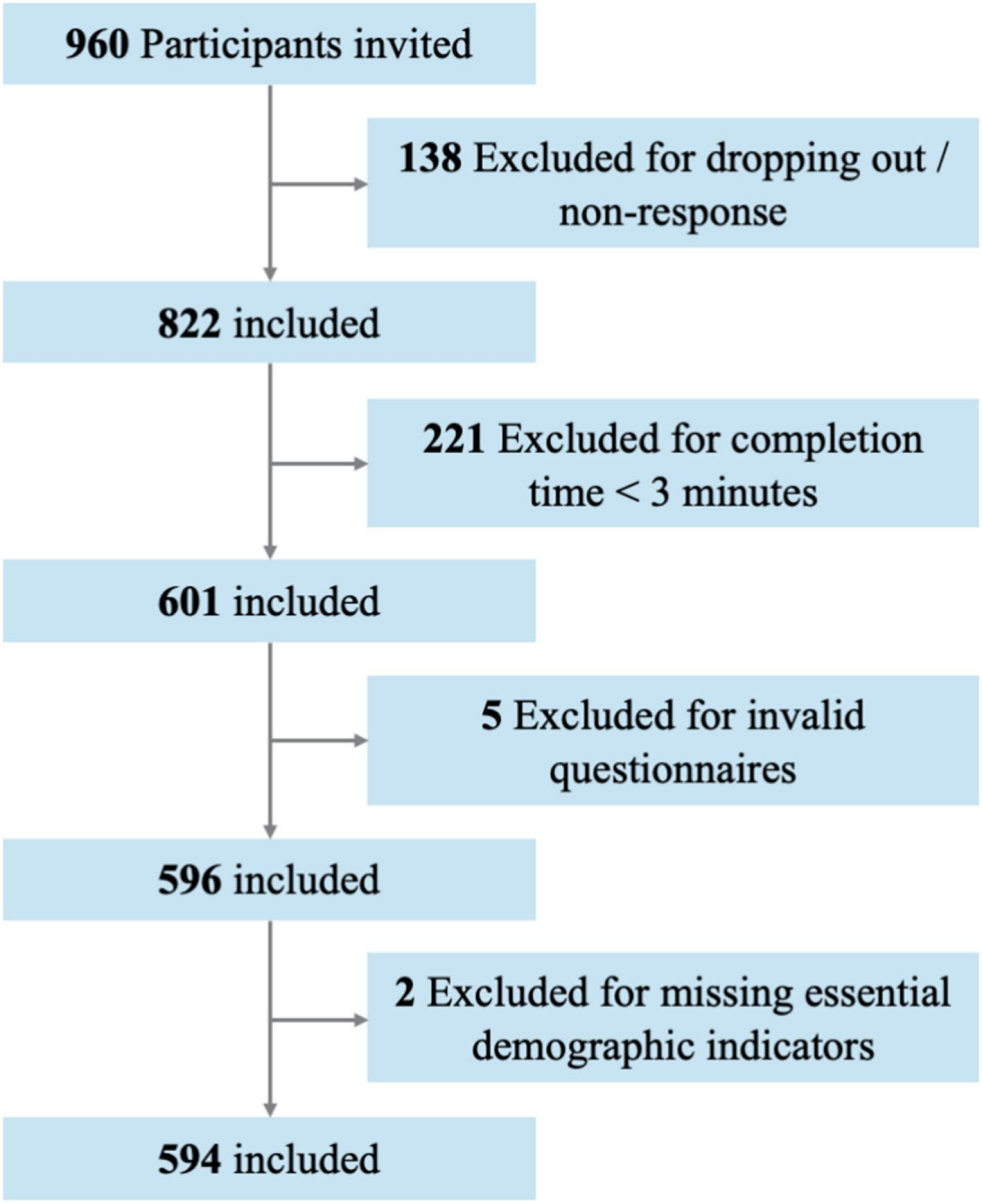
Participant recruitment and screening flow diagram.

Participants were eligible if they were full-time vocational college students, were able to read and complete the questionnaire in Chinese independently, and provided voluntary informed consent. Students were excluded if they self-reported a severe physical illness or psychiatric disorder that could affect questionnaire completion, or if they were concurrently participating in another health-related study. A total of 960 students were invited to participate in this survey. Among them, 822 questionnaires were returned, with a response rate of 85.6%. Of these, 228 responses were excluded due to completion time less than 3 min (*n* = 221) and missing essential indicators (*n* = 7). A total of 594 students were included in the final analysis. A participant flow diagram is presented in Figure 1.

### 2.4 Sample size

Schumacker and Lomax (23) recommended 250–500 participants for mediation analysis. In addition, Meyer and Morin (24) suggested that LPA generally achieves stable and interpretable results with sample sizes ranging from 200 to 500, whereas sample size larger than 500 may yield more robust results. Accordingly, the final sample size in the present study was considered adequate for the planned analyses.

### 2.5 Data collection

Data were collected using an anonymous online questionnaire distributed via class instructors or student representatives. Based on previous studies (6, 25), a self-designed questionnaire, including demographic information and measures of eHealth literacy, health self-efficacy, and health-promoting lifestyle, was used to collect data.

#### 2.5.1 Demographic variables

The demographic information included gender, academic year, major type, place of origin, family economic status, parental education level, and self-rated health. Major type was categorized into two groups based on students’ academic disciplines: Science and Engineering, and Arts and Humanities.

#### 2.5.2 eHL

The eHL was assessed using the Simplified Chinese version of the eHealth Literacy Scale (SC-eHEALS). The original eHEALS was developed by Norman et al. in 2006 to evaluate individuals’ perceived skills to find, evaluate, and apply web-based health information to manage health issues (26). Xu et al. (27) revised and validated the simplified Chinese version through rigorous cross-cultural adaptation and psychometric testing, demonstrating satisfactory reliability and validity in Chinese populations. The SC-eHEALS consists of eight items, each rated on a 5-point Likert scale (1 = strongly disagree, 5 = strongly agree), with total scores ranging from eight to 40. Higher scores indicate better perceived eHL. Previous studies have commonly used a total score of 32 or higher to indicate high level of eHL. In this study, the Cronbach’s *α* coefficient for the SC-eHEALS was 0.821, indicating good internal consistency.

#### 2.5.3 HSE

The HSE was measured using the Self-Rated Abilities for Health Practices–Adolescent Version (SRAHP-A), developed by Chilton et al. (28) to assess adolescents’ perceived self-efficacy for health-promoting behaviors. The scale is grounded in self-efficacy theory and health promotion theory, which are consistent with the aims of this study. The SRAHP-A consists of 28 items across four domains: nutrition (six items), exercise (seven items), responsible health practices (seven items), and psychological well-being (eight items). Each item is rated on a 5-point Likert scale (0 = not at all, 4 = completely), with total scores ranging from zero to 112. Higher scores reflect stronger perceived HSE. The scale has been used in multiple studies and has demonstrated satisfactory reliability and validity (29, 30). In this study, the Cronbach’s *α* coefficient for the total scale was 0.968, with subscale coefficients of 0.898 for nutrition, 0.886 for exercise, 0.899 for responsible health practices, and 0.939 for psychological well-being, indicating excellent internal consistency.

#### 2.5.4 HPL

The Health-Promoting Lifestyle Profile II (HPLP-II) was used to assess HPL. The original scale was developed by Walker et al. (31). This study used the mainland China–adapted version validated by Cao et al. (32), which contains 40 items across six domains: interpersonal relations (five items), health responsibility (11 items), stress management (five items), nutrition (six items), physical activity (eight items), and spiritual growth (five items). Responses are rated on a 4-point Likert scale (1 = never, 4 = routinely), with total scores ranging from 40 to 160. Higher scores indicate a healthier lifestyle. In this study, the Cronbach’s *α* coefficient for the total scale was 0.970, with subscale coefficients of 0.929 for interpersonal relations, 0.929 for health responsibility, 0.907 for stress management, 0.884 for nutrition, 0.924 for physical activity, and 0.916 for SG.

### 2.6 Quality control

To ensure data quality, a pilot survey was conducted with 30 students to evaluate item clarity and study feasibility. The average completion time was 6 min, and minor wording revisions were made based on the pilot results. In the formal survey, responses were excluded if completion time was less than 3 min (pre-specified based on pilot study) or if they contained missing data on key variables, including the primary outcome (eHealth literacy scale) and essential demographic characteristics. To encourage participation, all participants received a box of surgical masks and were eligible for a random prize draw.

Because the core variables in this study (e.g., eHL, HSE, and HPL) were measured simultaneously using self-report questionnaires, common method bias was considered a potential methodological concern. Several procedural measures were implemented to minimize this risk, including anonymous participation, voluntary responses, and standardized survey administration procedures. In addition, Harman’s single-factor test was performed via exploratory factor analysis to examine the extent of common method bias (33). Consistent with previous studies, severe common method bias was considered present if the first factor explained more than 40% of the total variance (34). In the present study, the first unrotated factor accounted for 37.72% of the total variance, suggesting that common method bias was unlikely to have substantially affected the study findings, although its presence cannot be completely ruled out.

Two sensitivity analyses were conducted to examine the robustness of the findings. First, latent profile analyses were re-estimated using the full sample to assess the stability of the identified profiles. Second, mediation models were re-run with additional adjustment for age, place of origin, parental education, and major type.

This study is reported following the guideline of Strengthening the Reporting of Observational Studies in Epidemiology (STROBE).

### 2.7 Data analysis

All statistical analyses were conducted using SPSS 26.0 and Mplus 8.3. Descriptive statistics were used to summarize participants’ demographic characteristics and the main study variables. Reliability analysis of the used scales and correlation analysis between variables were then performed.

The LPA was conducted to identify eHL profiles among vocational college students. In the LPA, models with one to five profiles were estimated. Item means were freely estimated across profiles, whereas item variances were held equal across profiles. The optimal model was selected based on multiple model fit indices, including the Akaike Information Criterion (AIC), Bayesian Information Criterion (BIC), and the adjusted Bayesian Information Criterion (aBIC) (35). Lower values of AIC, BIC, and aBIC indicate better model fit (36). Entropy value was used to assess classification accuracy, with values greater than 0.8 indicating good classification accuracy. Model selection also considered the practical significance and interpretability of each solution. In addition, each profile was required to account for at least 5% of the total sample (37). Average posterior probabilities were used to assess classification quality. Local independence was evaluated by examining residual correlations among indicators.

Mediation analysis was performed using the PROCESS macro (Model 4) to examine the mediating role of HSE in the association between eHL profiles and HPL. eHL profile membership was entered as a categorical independent variable, with the Low Application–High Confidence profile specified as the reference group. Direct, indirect, and total effects were estimated using a bias-corrected bootstrap method with 5,000 resamples, and 95% confidence intervals (CIs) were calculated. If the 95% CI did not contain zero, the corresponding effect was considered statistically significant.

The mediation model consisted of three regression equations: (1) HPL was regressed on eHL profiles to estimate the total effect; (2) HSE was regressed on eHL profiles to estimate the association between eHL profiles and the mediator; and (3) HPL was regressed on both eHL profiles and HSE to estimate the direct and indirect effects. Covariate selection was guided primarily by theoretical relevance and prior empirical evidence, with preliminary univariate analyses used only as supplementary information (6, 25, 38). The same demographic covariates were included in all three regression models to minimize potential confounding.

## 3 Results

### 3.1 Sample characteristics

After excluding invalid questionnaires, a final sample of 594 students was included in the analysis. No significant differences in demographic characteristics were observed between included and excluded participants (all *p* > 0.05). The final sample consisted of 594 students. The mean age was 19.8 ± 1.6 years, ranging from 16 to 25 years. Gender, family economic status, and self-rated health were significantly associated with HPL (*p* < 0.05). Table 1 shows more detailed demographic characteristics of participants.

**Table 1:** Demographic characteristics of participants (*N* = 594).

Variables	*N* (%)	Outcome variable: HPL	
		(Mean ± SD)	*P*
Gender			<0.001
Male	340 (57.2%)	119.12 ± 24.70	
Female	254 (42.8%)	111.07 ± 23.42	
Academic Year			0.992
Freshman	283 (47.6%)	115.62 ± 25.05	
Sophomore	179 (30.1%)	114.06 ± 23.70	
Junior	132 (22.3%)	118.01 ± 23.82	
Major Type			0.577
Science and Engineering	477 (80.3%)	115.96 ± 24.64	
Arts and Humanities	117 (19.7%)	114.55 ± 23.82	
Place of Origin			0.162
Urban	198 (33.3%)	117.67 ± 25.32	
Rural	396 (66.7%)	114.69 ± 24.00	
Family Economic Status			0.002
Below average	80 (13.5%)	108.60 ± 22.35	
Average	474 (79.8%)	116.11 ± 24.35	
Above average	40 (6.7%)	124.78 ± 26.61	
Father’s Educational Level			0.175
Vocational college or below	457 (80.0%)	115.00 ± 23.59	
Bachelor’s degree or above	119 (20%)	118.40 ± 27.64	
Mother’s Educational Level			0.583
Vocational college or below	528 (88.9%)	115.48 ± 24.10	
Bachelor’s degree or above	66 (11.1%)	117.24 ± 28.02	
Self-rated Health			<0.001
Poor/Fair	39 (6.6%)	107.92 ± 22.46	
Good	368 (62.0%)	111.78 ± 22.56	
Excellent	187 (31.5%)	124.98 ± 25.91	

### 3.2 Descriptive statistics

The mean total scores for eHL, HSE, and HPL were 25.21 ± 5.26, 81.33 ± 20.12, and 115.68 ± 24.47, respectively. The mean eHL score was below the commonly used threshold of 32 for high eHL. Among the HSE dimensions, psychological well-being showed the highest item mean score (23.58 ± 6.38), whereas nutrition showed the lowest item mean score (17.53 ± 4.66). Among the HPL dimensions, health responsibility had the highest item mean score (29.95 ± 7.98), while spiritual growth had the lowest item mean score (15.01 ± 3.88). Detailed descriptive statistics are presented in Table 2.

**Table 2:** Descriptive statistics of eHL, HSE, and HPL.

Variables	Possible range	$\bar{x}\pm$ SD
eHL total score	8–40	25.21 ± 5.26
HSE total score	0–112	81.33 ± 20.12
Exercise (HSE)	0–28	20.02 ± 5.48
Nutrition (HSE)	0–24	17.53 ± 4.66
RHP (HSE)	0–28	20.19 ± 5.58
PW (HSE)	0–32	23.58 ± 6.38
HPL total score	40–160	115.68 ± 24.47
IR (HPL)	5–20	16.13 ± 3.66
HR (HPL)	11–44	29.95 ± 7.98
SM (HPL)	5–20	15.09 ± 3.51
Nutrition (HPL)	6–24	17.89 ± 4.14
PA (HPL)	8–32	21.61 ± 6.14
SG (HPL)	5–20	15.01 ± 3.88

RHP, responsible health practices; PW, psychological well-being; IR, interpersonal relations; HR, health responsibility; SM, stress management; PA, physical activity; SG, spiritual growth.

### 3.3 Correlation analysis results

As presented in Table 3, correlation analysis showed that total scores of the three main variables were significantly positively correlated with each other. Specifically, the eHL total score was positively associated with both the HSE total score (*r* = 0.46, *p* < 0.01) and the HPL total score (*r* = 0.35, *p* < 0.01). Additionally, a strong positive correlation was observed between the HSE total score and the HPL total score (*r* = 0.64, *p* < 0.01).

**Table 3:** Pearson correlation analysis of eHL, HSE, and HPL.

Variables	1	2	3	4	5	6	7	8	9	10	11	12	13
eHL total score	1												
HSE total score	0.461**	1											
Exercise (HSE)	0.427**	0.880**	1										
Nutrition (HSE)	0.434**	0.908**	0.746**	1									
RHP (HSE)	0.449**	0.925**	0.730**	0.813**	1								
PW (HSE)	0.379**	0.925**	0.734**	0.780**	0.821**	1							
HPL total score	0.352**	0.639**	0.542**	0.579**	0.546**	0.651**	1						
IR (HPL)	0.305**	0.469**	0.385**	0.431**	0.398**	0.485**	0.742**	1					
HR (HPL)	0.324**	0.559**	0.481**	0.505**	0.487**	0.556**	0.884**	0.552**	1				
SM (HPL)	0.250**	0.557**	0.450**	0.497**	0.492**	0.575**	0.824**	0.595**	0.697**	1			
Nutrition (HPL)	0.260**	0.543**	0.438**	0.490**	0.474**	0.562**	0.858**	0.644**	0.680**	0.720**	1		
PA (HPL)	0.298**	0.515**	0.445**	0.474**	0.426**	0.524**	0.838**	0.489**	0.671**	0.582**	0.646**	1	
SG (HPL)	0.293**	0.541**	0.489**	0.479**	0.440**	0.550**	0.799**	0.600**	0.579**	0.609**	0.667**	0.645**	1

RHP, responsible health practices; PW, psychological well-being; IR, interpersonal relations; HR, health responsibility; SM, stress management; PA, physical activity; SG, spiritual growth; **p < 0.01.

### 3.4 Results of latent profiles analysis of eHL

As shown in Table 4, the AIC, BIC, and aBIC values decreased as the number of profiles increased. Considering model fit indices, entropy values, profile proportions, statistical tests, and interpretability, the three-profile solution was selected as the optimal model. Compared with the two-profile solution, the three-profile model demonstrated higher classification accuracy (Entropy = 0.912) and more balanced profile proportions. Although the four-profile solution showed slightly better fit indices, the additional profile appeared to reflect a subdivision of an existing profile and did not yield meaningful practical distinctions. In the five-profile solution, the LMR test was not statistically significant (*p* = 0.056). The average posterior probabilities for the three-profile model were 0.984, 0.967 and 0.940, respectively, all exceeding 0.80, indicating satisfactory classification accuracy (Supplementary Table S1). No residual correlations exceeded |0.10|, supporting the assumption of local independence. Furthermore, sensitivity analysis using the full sample (*N* = 815) confirmed that the three-profile solution remained optimal (Supplementary Table S2).

**Table 4:** Fit statistics for latent profile models of eHL.

Profiles	AIC	BIC	aBIC	Entropy	LMRT(*p*)	BLRT(*p*)	Profile Proportions
1-Profile	13300.482	13370.672	13319.877				1
2-Profile	12297.947	12407.619	12328.251	0.881	<0.001	<0.001	0.638/0.362
3-Profile	11587.648	11736.802	11628.863	0.912	<0.001	<0.001	0.152/0.520/0.328
4-Profile	11504.333	11692.969	11556.457	0.877	0.003	<0.001	0.150/0.485/0.258/0.108
5-Profile	11476.453	11704.571	11539.487	0.841	0.056	<0.001	0.153/0.412/0.091/0.254/0.089

**Figure 2:**
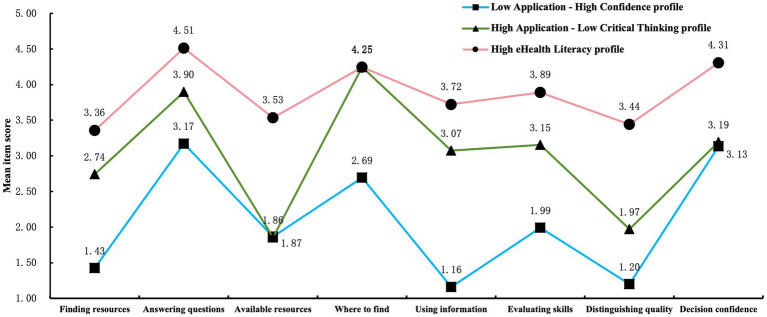
Map of eHL categories of the participants.

Based on the LPA and the response patterns illustrated in Figure 2, the three profiles were labeled to reflect their distinct eHL patterns. Profile 1 (*n* = 89, 15%), labeled Low Application-High Confidence, was characterized by low scores on Finding resources, Available resources, and Using information (all < 2.0) and relatively high scores on Decision confidence and Answering questions (all > 3.0). Profile 2 (*n* = 309, 52%), labeled High Application-Low Critical Thinking, demonstrated high scores on Where to find (4.25) and Answering questions (3.90) but a notably low score on Distinguishing quality (1.97). Profile 3 (*n* = 196, 33%), labeled High eHL, exhibited consistently high scores across all items, with mean item scores ranging from 3.36 to 4.51.

### 3.5 Differences in HSE and HPL across eHL profiles

Table 5 shows the significant differences in HSE total scores (*F*[2,591] = 67.067, *p* < 0.001) and HPL total scores (Welch’s *F*[2,564] = 59.739, *p* < 0.001) across the three identified eHL profiles. Specifically, HSE total scores increased stepwise across the three eHL profiles, with Profile 3 showing the highest scores, followed by Profile 2 and Profile 1; all pairwise comparisons were statistically significant (all *p* < 0.05). Regarding HPL, students in Profile 2 and Profile 3 exhibited significantly higher HPL total scores than those in Profile 1 (*p* < 0.001), whereas no significant difference was found between Profile 2 and Profile 3 (*p* > 0.05).

**Table 5:** Comparison of eHL, HSE, and HPL scores across the three identified eHL profiles.

Variables	Profile 1 (*n* = 89)	Profile 2 (*n* = 309)	Profile 3 (*n* = 196)	*F*/Welch’s *F*	*Post hoc* tests
eHL total score^b^	16.61 ± 1.73	24.07 ± 2.05	30.98 ± 2.55	1414.31**	1 < 2 < 3
HSE total score^b^	63.12 ± 16.89	81.17 ± 18.64	89.97 ± 18.06	67.067**	1 < 2 < 3
HPL total score^a^	91.98 ± 26.52	119.55 ± 22.81	120.49 ± 19.33	59.739**	1 < 2, 1 < 3

^a^Welch’s *F* test; ^b^one-way ANOVA; **P < 0.01.

### 3.6 Mediation effect of HSE

Gender, academic year, family economic status, and self-rated health were included as covariates in all mediation models to control for potential confounding. With the Low Application–High Confidence profile as the reference group, both the High Application–Low Critical Thinking profile (*β* = 1.062, *p* < 0.001) and the High eHL profile (*β* = 1.083, *p* < 0.001) were positively associated with HPL in Model 1. Both profiles were also positively associated with HSE in Model 2 (*R^2^* = 0.279, *p* < 0.001; Table 6). After HSE was included in the model, the associations between eHL profiles and HPL were weakened but remained statistically significant. The regression coefficients decreased to 0.599 for the High Application–Low Critical Thinking profile and 0.386 for the High eHL profile (Table 6).

**Table 6:** The mediating effect of HSE on HPL.

Models	Variables	*R* ^2^	*F*_(df)_	*β*	*t*
Model 1	Dependent variable: HPL	0.245	31.757 _(6)_		
	High Application – Low Critical Thinking			1.062	10.014***
	High eHealth Literacy			1.083	9.584***
Model 2	Dependent variable: HSE	0.279	37.847 _(6)_		
	High Application – Low Critical Thinking			0.837	8.082***
	High eHealth Literacy			1.260	11.414***
Model 3	Dependent variable: HPL	0.466	72.923 _(7)_		
	High Application – Low Critical Thinking			0.599	6.360***
	High eHealth Literacy			0.386	3.669***
	Health Self-efficacy			0.553	15.549***

*β* values are standardized coefficients. eHL profile membership was entered as a categorical variable, with the Low Application–High Confidence profile serving as the reference category. All models were adjusted for gender, academic year, family economic status, and self-rated health. ***p < 0.001.

**Figure 3:**
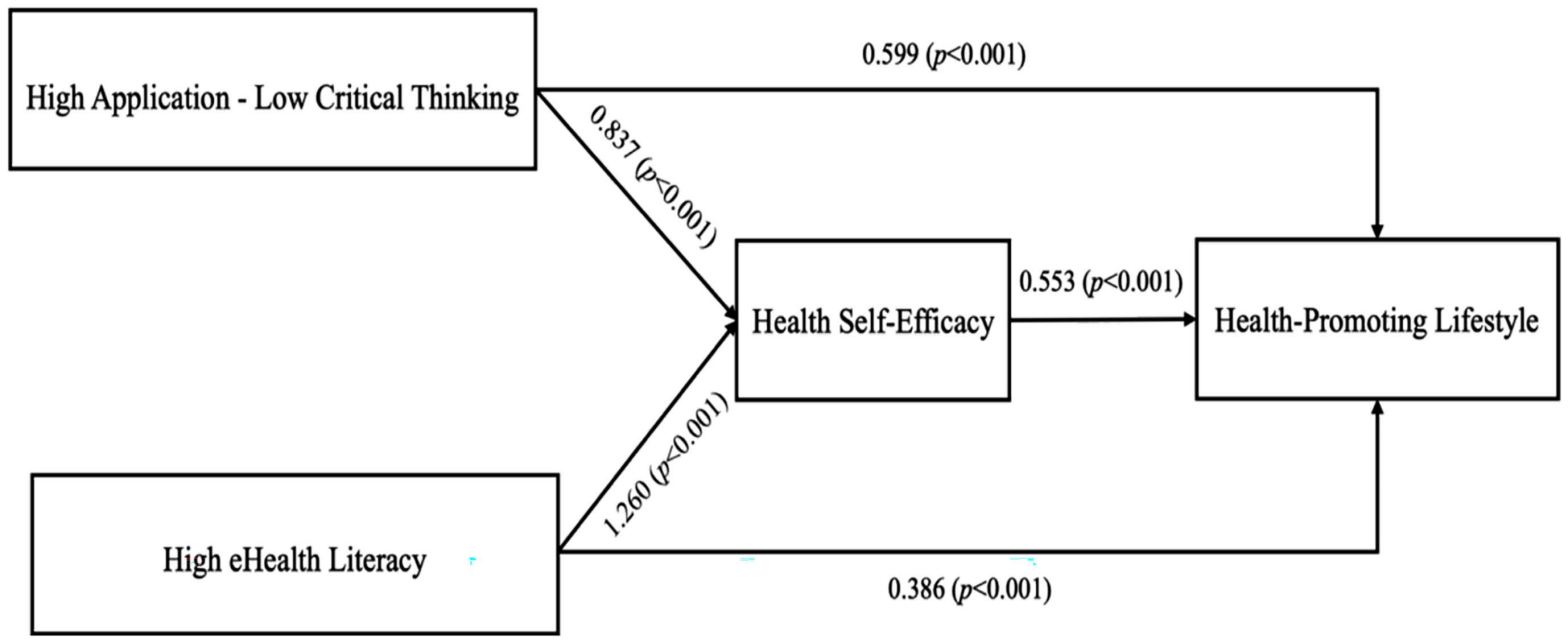
The mediating effect of HSE between eHL profiles and HPL.

As shown in Figure 3 and Table 7, the indirect effect of HSE for the High Application–Low Critical Thinking profile was 0.463 (95% CI: 0.345–0.586), accounting for 43.62% of the total effect. For the High eHL profile, the indirect effect was 0.697 (95% CI: 0.560–0.840), accounting for 64.37% of the total effect. The 95% CIs for both indirect effects did not include zero. Sensitivity analyses with additional adjustment for age, place of origin, parental education, and major type yielded consistent results (Supplementary Tables S3, S4), indicating robustness of the findings.

**Table 7:** Direct and indirect effects of HSE on HPL.

Path	Effect	Boot SE	Boot LLCI	Boot ULCI	Relative Effect (%)
Path 1: “High Application – Low Critical Thinking” profile → self-efficacy → HPL					
Direct Effect	0.599	0.124	0.350	0.836	56.38%
Indirect Effect	0.463	0.062	0.345	0.586	43.62%
Total Effect	1.062	0.106	0.853	1.270	
Path 2: “High eHealth Literacy” profile → self-efficacy → HPL					
Direct Effect	0.386	0.129	0.127	0.632	35.63%
Indirect Effect	0.697	0.072	0.560	0.840	64.37%
Total Effect	1.083	0.113	0.861	1.305	

## 4 Discussion

To our knowledge, this is the first study to apply LPA to identify distinct eHL profiles in vocational college students. This study emphasized the heterogeneity of eHL in this population, which is consistent with the findings of Stellefson et al. (18). Through LPA, three profiles were identified: Low Application – High Confidence profile (Profile 1, 15%), High Application – Low Critical Thinking profile (Profile 2, 52%) and High eHL profile (Profile 3, 33%). Significant differences were observed in HPL and HSE across the three eHL profiles. HSE was statistically tested as a mediator between eHL profiles and HPL.

Furthermore, in this study, the mean total score of eHL (25.21) was below the threshold of 26, which has been used to indicate a relatively low level of eHL (39). This score was also lower than the reported averages among students in general universities in China (25) and those reported in several European countries (6). Together, these findings suggest that vocational college students may represent a digitally disadvantaged subgroup and warrant targeted attention in efforts to promote health equity.

Students in the Low Application – High Confidence profile were characterized by high decision confidence despite limited practical digital skills. This pattern may reflect overconfidence and is consistent with patterns described in the Dunning–Kruger literature. Specifically, individuals with lower levels of competence may overestimate their abilities because they have limited awareness of their own knowledge gaps and performance limitations (40). Consistent with this finding, Bertolazzi et al. (41) identified overconfidence in online health information seeking among university students, which may lead to irrational self-care behaviors. Vocational college students may possess intermediate levels of health-related knowledge, a stage at which overconfidence has been shown to peak (42). Moreover, this bias may be further reinforced by algorithm-driven personalization, which could increase exposure to fragmented, homogeneous and low-quality health information (43, 44). Therefore, interventions for this group should shift from general motivational encouragement to more rigorous training in information verification and digital competencies.

Turning to Profile 2, which represented the largest subgroup (52%), these students demonstrated strong skills in locating resources and answering questions but had difficulties in critically evaluating the quality of health information. This imbalance may stem from the vocational education environment, where practical skills are emphasized over the development of critical thinking (45). In addition, low HSE may hinder students’ confidence in their ability to evaluate health information (43, 46). Furthermore, individuals with limited eHL may be more likely to encounter lower-quality or misleading health information online (47, 48). As a result, students in this group may be particularly vulnerable to pervasive misinformation and disinformation. Accordingly, digital health interventions for this group should not only teach students how to find health information but also strengthen their ability to critically evaluate health information on digital platforms.

Finally, Profile 3 (33%, High eHL) exhibited consistently strong performance across application, information evaluation, and decision-making dimensions. Students in this profile demonstrated relatively strong perceived eHealth literacy across all assessed domains. Although this pattern is broadly consistent with aspects of critical eHL proposed by Nutbeam (49), eHEALS does not fully capture this construct; therefore, this interpretation should be made with caution. At this stage, the value of eHL may extend beyond individual health behaviors to broader population-level benefits (50). Notably, in the current digital landscape, a trust paradox has emerged: while scientifically rigorous institutions face increasing skepticism, unvetted social media influencers are often perceived as more credible. A strategic response to this challenge is to emphasize co-production in the design and delivery of digital health initiatives from the outset (3, 51). Therefore, students in this profile may represent a valuable resource for peer-led digital health promotion initiatives. Interventions targeting these students could empower them to act as peer educators and co-creators in digital health, potentially facilitating the dissemination of reliable health information among their peers (52).

A particularly notable finding of this study is that HPL total scores in Profile 3 did not significantly differ from those in Profile 2. This pattern suggests a ceiling effect: improvements in eHL beyond a certain threshold may not lead to further gains in HPL. One plausible explanation is that eHL acts as a midstream enabling factor rather than a panacea for health behavior (50). Healthy lifestyles are shaped not only by individual digital skills but also by broader structural factors, including social, cultural, environmental, and economic influences, which are often underemphasized in intervention studies (53). Indeed, environmental and policy-related factors have been shown to exert stronger influences on college students’ eHL than individual-level demographic characteristics (54).

From the perspective of Social Cognitive Theory, health behavior is influenced not only by digital competencies but also by cognitive mediators such as HSE (19, 46). Our mediation analysis further revealed that the relative contribution of HSE differed between Profile 2 and Profile 3, even though their HPL total scores did not differ significantly. Higher overall eHL in Profile 3 was associated with a stronger mediating role of HSE, suggesting that enhanced digital literacy may promote healthier practices partly through greater psychological empowerment. Although causal relationships cannot be fully established due to the cross-sectional design, the findings support the potential role of HSE as an important psychological pathway linking eHL and HPL. Importantly, although HSE and HPL share some common behavioral domains, they represent distinct constructs, reflecting confidence in engaging in health-promoting behaviors and the actual adoption of those behaviors, respectively. Thus, students with distinct eHL profiles may show similar levels of HPL while differing in the psychological mechanisms underlying these behaviors. These findings highlight the importance of combining individual capability building with broader environmental support, rather than focusing solely on individual factors.

Overall, the three identified profiles reveal heterogeneity among vocational college students in both overall eHL and the configuration of subdomain competencies. For students in the Low Application–High Confidence profile, targeted interventions should prioritize practical digital health skills training and awareness of individual knowledge gaps. Students in the High Application–Low Critical Thinking profile may benefit most from training in information appraisal and critical evaluation. In contrast, students in the High eHL profile may be well positioned to participate in peer-led health promotion and digital health co-creation initiatives. These findings suggest that a one-size-fits-all approach may be insufficient and that profile-specific intervention strategies could be more effective in promoting healthy lifestyles among vocational college students.

### 4.1 Strengths and limitations

This study has several strengths. First, to our knowledge, it is the first study to identify distinct eHL profiles among vocational college students using LPA, which captures the heterogeneity of eHL in this population. Second, grounded in Social Cognitive Theory, it further clarifies the mediating role of HSE in the pathway from eHL profiles to HPL. Third, the identification of a ceiling effect highlights the importance of environmental and systemic factors, and the proposed tailored intervention strategies for different subgroups may offer practical implications for digital health education.

However, several limitations should be acknowledged when interpreting these findings. First, the cross-sectional design limits causal inference, particularly regarding the observed mediation effects. Future longitudinal, multicenter studies with objective measures are therefore warranted. Second, all variables were measured using self-report questionnaires, which may introduce recall bias and common method variance. Although Harman’s single-factor test did not indicate severe common method bias, this possibility cannot be completely ruled out. Third, the study was conducted in a single university in China, and participation was voluntary, which may limit generalizability and introduce self-selection bias. In addition, potential class-level clustering effects were not explicitly accounted for, which may have led to underestimation of standard errors. Finally, although data cleaning procedures were implemented to improve data quality, the absence of embedded attention-check items may have limited the ability to identify insufficient-effort responses.

## 5 Conclusion

This study used a person-centered approach to identify different eHL profiles in vocational college students and to examine the mediating role of HSE between eHL and HPL. The findings indicate that vocational college students may represent a digitally disadvantaged subgroup within the broader university population and that their eHL is substantially heterogeneous. Three profiles were identified, and each profile showed different levels of health-promoting behaviors and HSE. Importantly, HSE was statistically verified as a significant mediator in the association between eHL profiles and HPL. The findings highlight the importance of tailoring digital health education and health promotion interventions to the specific needs of different eHL subgroups. However, causal inference remains limited by the cross-sectional design.

## Data Availability

The raw data supporting the conclusions of this article will be made available by the authors, without undue reservation.
