# Supplementary material for: eHealth literacy, health self-efficacy, and health-promoting lifestyle among vocational college students: a latent profile and mediation analysis study
Source: Front Public Health. 2026 Jul 15;14:1864980. doi: 10.3389/fpubh.2026.1864980 (PMC13415940; doi:10.3389/fpubh.2026.1864980)
Supplement: Supplementary file 1 [file Table_1.docx]

**Supplementary Table S1.** Average posterior probabilities for each latent profile

| **Profile** | **Profile 1** | **Profile 2** | **Profile 3** |
| --- | --- | --- | --- |
| P1 | 0.984 | 0.016 | 0.000 |
| P2 | 0.005 | 0.967 | 0.028 |
| P3 | 0.000 | 0.060 | 0.940 |

Note: Values on the diagonal represent classification accuracy for each profile.
