# Supplementary material for: eHealth literacy, health self-efficacy, and health-promoting lifestyle among vocational college students: a latent profile and mediation analysis study
Source: Front Public Health. 2026 Jul 15;14:1864980. doi: 10.3389/fpubh.2026.1864980 (PMC13415940; doi:10.3389/fpubh.2026.1864980)
Supplement: Supplementary file 2 [file Table_2.docx]

**Supplementary Table S2.** Sensitivity analysis of latent profile models based on the full sample after excluding missing indicators (N = 815).

| **Profiles** | **AIC** | **BIC** | **aBIC** | **Entropy** | **LMRT** | **BLRT** | **Proportion** |
| --- | --- | --- | --- | --- | --- | --- | --- |
| 1-profile | 19094.427 | 19169.814 | 19119.004 |  |  |  | 1 |
| 2-profile | 17991.748 | 18109.542 | 18030.151 | 0.851 | ＜0.001 | ＜0.001 | 0.633/0.367 |
| 3-profile | 17345.438 | 17505.638 | 17397.666 | 0.877 | ＜0.001 | ＜0.001 | 0.519/0.154/0.327 |
| 4-profile | 17162.303 | 17364.908 | 17228.356 | 0.907 | 0.238 | ＜0.001 | 0.151/0.512/0.309/0.028 |
| 5-profile | 17075.484 | 17320.495 | 17155.362 | 0.925 | 0.390 | ＜0.001 | 0.018/0.153/0.508/0.309/0.013 |
