# Supplementary material for: eHealth literacy, health self-efficacy, and health-promoting lifestyle among vocational college students: a latent profile and mediation analysis study
Source: Front Public Health. 2026 Jul 15;14:1864980. doi: 10.3389/fpubh.2026.1864980 (PMC13415940; doi:10.3389/fpubh.2026.1864980)
Supplement: Supplementary file 3 [file Table_3.docx]

**Supplementary Table S3.** Sensitivity analysis of mediation models with additional adjustment for age, place of origin, parental education, and major type.

| **Models** | **Variables** | **R^2^** | **F_(df)_** | **β** | **t** |
| --- | --- | --- | --- | --- | --- |
| **Model 1** | **Dependent variable: HPL** | 0.251 | 17.747_（11）_ |  |  |
|  | High Application - Low Critical Thinking |  |  | 1.054 | 9.926*** |
|  | High eHealth Literacy |  |  | 1.079 | 9.478*** |
| **Model 2** | **Dependent variable: HSE** | 0.29 | 21.643_（11）_ |  |  |
|  | High Application - Low Critical Thinking |  |  | 0.835 | 8.073*** |
|  | High eHealth Literacy |  |  | 1.267 | 11.434*** |
| **Model 3** | **Dependent variable: HPL** | 0.47 | 42.870_（12）_ |  |  |
|  | High Application - Low Critical Thinking |  |  | 0.591 | 6.266*** |
|  | High eHealth Literacy |  |  | 0.376 | 3.543*** |
|  | Health Self-efficacy |  |  | 0.555 | 15.469*** |

Note: All models were adjusted for gender, academic year, family economic status, self-rated health, age, place of origin, parental education, and major type. ****P* < 0.001.
