# Supplementary material for: eHealth literacy, health self-efficacy, and health-promoting lifestyle among vocational college students: a latent profile and mediation analysis study
Source: Front Public Health. 2026 Jul 15;14:1864980. doi: 10.3389/fpubh.2026.1864980 (PMC13415940; doi:10.3389/fpubh.2026.1864980)
Supplement: Supplementary file 4 [file Table_4.docx]

**Supplementary Table S4.** Robustness checks of direct, indirect and total effects in mediation models under alternative covariate specifications.

| **Path** | **Effect** | **Boot SE** | **Boot LLCI** | **Boot ULCI** | **Relative Effect (%)** |
| --- | --- | --- | --- | --- | --- |
| **Path 1: “High Application - Low Critical Thinking” profile →** **self-efficacy → HPL** | | | | | |
| **Direct Effect** | 0.591 | 0.094 | 0.406 | 0.776 | 56.07% |
| **Indirect Effect** | 0.463 | 0.06 | 0.346 | 0.582 | 43.93% |
| **Total Effect** | 1.054 | 0.106 | 0.846 | 1.263 |  |
| **Path 2: “High eHealth Literacy” profile →self-efficacy → HPL** | | | | | |
| **Direct Effect** | 0.376 | 0.106 | 0.168 | 0.584 | 34.84% |
| **Indirect Effect** | 0.703 | 0.073 | 0.562 | 0.845 | 65.16% |
| **Total Effect** | 1.079 | 0.114 | 0.855 | 1.302 |  |
